# Point cloud completion in challenging indoor scenarios with human motion

**DOI:** 10.3389/frobt.2023.1184614

**Published:** 2023-05-10

**Authors:** Chengsi Zhang, Stephen Czarnuch

**Affiliations:** ^1^ Department of Electrical and Computer Engineering, Faculty of Engineering and Applied Science, Memorial University of Newfoundland, St. John’s, NL, Canada; ^2^ Department of Electrical and Computer Engineering, Faculty of Engineering and Applied Science and the Discipline of Emergency Medicine, Faculty of Medicine, Memorial University of Newfoundland, St. John’s, NL, Canada

**Keywords:** 3D completion, point cloud registration, point cloud segmentation, 3D data analysis, 3D data processing

## Abstract

Combining and completing point cloud data from two or more sensors with arbitrarily relative perspectives in a dynamic, cluttered, and complex environment is challenging, especially when the two sensors have significant perspective differences while the large overlap ratio and feature-rich scene cannot be guaranteed. We create a novel approach targeting this challenging scenario by registering two camera captures in a time series with unknown perspectives and human movements to easily use our system in a real-life scene. In our approach, we first reduce the six unknowns of 3D point cloud completion to three by aligning the ground planes found by our previous perspective-independent 3D ground plane estimation algorithm. Subsequently, we use a histogram-based approach to identify and extract all the humans from each frame generating a three-dimensional (3D) human walking sequence in a time series. To enhance accuracy and performance, we convert 3D human walking sequences to lines by calculating the center of mass (CoM) point of each human body and connecting them. Finally, we match the walking paths in different data trials by minimizing the Fréchet distance between two walking paths and using 2D iterative closest point (ICP) to find the remaining three unknowns in the overall transformation matrix for the final alignment. Using this approach, we can successfully register the corresponding walking path of the human between the two cameras’ captures and estimate the transformation matrix between the two sensors.

## 1 Introduction

The use of three-dimensional (3D) data has grown dramatically in different industries, such as gaming (Yahav et al., 2007; Zhang, 2012), medicine (Haleem and Javaid, 2019; Ballard et al., 2020), and construction (Lin et al., 2019; Cui et al., 2020), and has become one of the most advanced data representation formats for research and commercial usage. Provided natively by modern 3D sensors, 3D data improve on 2D data (Herbert and Chen, 2015; Flusser et al., 2016) and are characterized by 3D geometric point coordinates and associated color attributes (Pereira et al., 2020; Raj et al., 2020; Tölgyessy et al., 2021). These 3D data representations have contributed to significant enhancements in the accuracy and reliability of various industrial applications (Herbert and Chen, 2015; Ballard et al., 2020; Fernandes et al., 2021). In addition, the processing and transferring time of the 3D datasets, which are generally quite large, are more practical now due to advances in modern computer hardware and networks. Point clouds have emerged as one of the primary data formats for 3D data because they can be directly captured by commercially available 3D sensors, such as depth (e.g., Microsoft Kinect) or light detection and ranging (LiDAR; e.g., Velodyne LiDAR) sensors. Therefore, point clouds have witnessed increasingly widespread usage in both research and production. The current state-of-the-art point cloud processing focuses on semantic scene understanding, coding, and completion tasks (Camuffo et al., 2022). However, point cloud data are strongly impacted by the perspective of the sensor and are limited to the sensor’s line of sight and field of view (Cheng et al., 2018). 3D sensors directly measure the distance from an object to the sensor, and these sensors have a finite capture range varying from a few meters (e.g., depth sensors) to hundreds of meters (e.g., LiDAR). The density of the captured point cloud is inversely proportional to the distance, such that objects close to the sensor are represented by spatially dense points, whereas objects further from the sensor are more sparsely represented. Notably, these 3D sensors can only capture data from the portions of objects facing the sensor. The backside of objects and objects occluded by other objects closer to the sensor do not contribute data to the 3D point cloud. Therefore, point cloud completion is required to generate a larger and more complete 3D scene using modern 3D sensors. Point cloud completion is the process of estimating missing information or combining partial information captured from multiple sensors or multiple perspectives together (Wen et al., 2020), resulting in a more wholesome representation of the scene or object.

One of the most common point cloud completion processes is point cloud registration, which is an approach to estimating the transformation (three degrees of rotation and three directions of translation) between a pair of 3D point clouds captured from different locations or perspectives with a partially overlapping field of view. After transforming one of the point clouds, the two point clouds are merged into one global point cloud, leading to a larger, more complete scene. Therefore, point cloud registration is a fundamental process for all industries that require complete 3D point cloud data. A complete point cloud provides a more accurate and detailed digital representation of the real world and is commonly used in gaming (Kurabayashi et al., 2016), as well as inspection and visualization in construction (Wang and Kim, 2019). Notably, point clouds provide the ability to manipulate the model in full 3D post-acquisition. Full point cloud representations of hospital facilities, such as operating rooms or intensive care units, have been especially useful for improving the efficiency and accuracy of medical activities (Liu et al., 2018; Fischer et al., 2022), which is also the application scenario we are targeting. In this study, we define the challenging real-world indoor environments we are targeting as scenes that are full of objects (i.e., cluttered), contain moving objects (i.e., dynamic), and involve different interactive activities (i.e., complex), such that the multiple sensors cannot be placed to ensure significant overlapping fields of view (FOV), and objects with rich and descriptive shape and texture are not guaranteed to be included in the overlapping FOV, or such that the same scene can appear significantly different from unique sensor perspectives.

The extensive usage of point clouds and point cloud completion in commercial industries has seen the rapid emergence of research into different approaches (Zhang et al., 2020), predominantly characterized by the use of local feature descriptors (Deng et al., 2019), global feature descriptors (Chen et al., 2019), and no-correspondence approaches (Huang et al., 2020a). The local feature descriptor and global feature descriptor registration approaches are the traditional approaches for point cloud registration. With local and global features, the transformation matrix is found by finding and describing key points in two point cloud images, identifying correspondences based on the similarity of the descriptors of the key points, and then minimizing the error in correspondences (Chen et al., 2016). The local feature descriptor extracts distinct geometric information from small key point clusters (e.g., objects) visible in the image (Guo et al., 2016). However, as the local feature descriptors are created from small groups of key points, their accuracy highly depends on the selection of representatively discriminative key points out of all the available points in the image, motivating some work on application-specific point cloud data optimization through undescriptive data removal (Ebrahimi and Czarnuch, 2021). Overall, the local feature descriptor is considerably sensitive to noise and error in the point cloud image and is characterized by the dilemma between feature generalization (i.e., too many features) and low descriptiveness (i.e., too many signatures) (Yang et al., 2016). With respect to image registration, the use of local features normally requires a large, common, overlapping field of view of at least 30% (Huang et al., 2021) and relatively similar sensor perspectives to succeed. The global feature descriptor summarizes the valuable geometric information present in the entire point cloud image (Mortensen et al., 2005). The global feature descriptor is more robust to localized noise and error in the image, summarizing the feature information across the entire image rather than utilizing individual features (Chen et al., 2018). However, the registration of two images is less robust than with the local feature descriptor approaches due to incorrect matching and low correspondences (Gelfand et al., 2005), particularly when the images are captured from distinctly unique perspectives or do not share significant overlapping FOV. Therefore, traditional feature-based approaches, whether local or global, are not ideal for registering the 3D point cloud frames captured in our challenging indoor environments. With the growth of computing power, a new no-correspondence category of point cloud registration has recently emerged (Le et al., 2019; Huang et al., 2020a; Yang et al., 2020). Instead of matching the corresponding feature points between the images, the overall projection error is minimized based on the feature key point that the algorithm uses. Therefore, the effects of both noise and density differences are mitigated, which improves the accuracy and reliability of registration in cluttered or low-density data and significantly boosts the overall processing time. Despite these improvements compared to the traditional feature-based approaches, no-correspondence approaches perform equally poorly when registering two point cloud images that have significant perspective differences or small common FOV (Yang et al., 2020) due to the difficulty of optimizing the descriptiveness and generalization of the selected feature points. Hence, accurate registration of two point clouds captured in challenging environments from unknown and potentially significantly different perspectives or with a limited field of view overlap remains an active challenge.

In this study, we propose a point cloud completion approach for point clouds captured from multiple sensors with unknown locations and perspective differences within challenging indoor environments for human tracking and detection applications, environments that prohibit the use of existing point cloud completion approaches. Our algorithm only assumes that a human can be seen walking in consecutively captured frames within the common field of view of pairs of arbitrarily placed 3D sensors and that both sensors can detect the ground plane. Notably, the overlapping field of view only needs to be as large as possible to detect a common human within pairs of sensors. Furthermore, the ground plane does not need to be entirely visible, but it only needs to be sufficiently large to be detected, as in our previous work (Zhang and Czarnuch, 2020). We propose to first dynamically identify the ground plane to partially align pairs of point clouds and then create a unique spatiotemporal feature based on the movements of humans within the point cloud images. The advantage of our algorithm is that, under our limited assumptions, we can extract highly generalizable and descriptive features that are temporally sequenced within each sensor’s data, allowing multiple point clouds captured in real-world environments from sensors with unknown perspectives and small common FOV to be combined. This allows us to quickly generate and align features detected in multiple sensors and reliably combine real-life point cloud data without complex setup as long as at least one human is moving, which is a reasonable assertion for tasks related to human motion detection (Zhang and Cao, 2018) and indoor environment simulation (Fischer et al., 2022).

## 2 Literature review

The point cloud completion problem has been most commonly approached through estimation or registration (Sarode et al., 2019; Gojcic et al., 2020; Wang et al., 2020; Fu et al., 2021). Point cloud registration methods merge directly sensed data from two point clouds to make a more comprehensive and representative 3D dataset over a larger area. Registration approaches mainly use local and global features and, more recently, no-correspondence registration. Both local and global feature descriptors have numerous existing implementations. The following sections describe some of the most commonly used descriptors in public open-source libraries. Conversely, estimation approaches utilize known information about the scene, the features of complex objects, and the template of objects to generate missing data within the point cloud (Xu et al., 2021) [e.g., generating data for the far side of a human’s body using only the visible near side (Tagliasacchi et al., 2009)].

### 2.1 Local 3D features

Historically, one of the most widely used local feature descriptors for 3D data is the Fast Point Feature Histogram (FPFH) (Rusu et al., 2009), which is implemented in the Point Cloud Library (Rusu and Cousins, 2011). It first builds a simplified point feature histogram (SPFH) for each point in the 3D data image by constructing one histogram from the point and its neighbors along each dimension. Then, the 3D FPFH is built based on the weighted sum of the SPFH of a feature point and the SPFHs of the points in the feature point’s support region (Rusu et al., 2009). Another commonly used local 3D feature descriptor is the Signature of Histogram of Orientations (SHOT) (Tombari et al., 2010), which is created by combining 3D Local Reference Frames (LRFs) (Tombari et al., 2010). The 3D LRF is constructed from each key point *k* and its neighboring points *np* as its support region by calculating the sum of the angle differences between the 3D normal vector of *k* and the 3D normal vector of *np*s in each local histogram bin after dividing the support region along the radial, azimuth, and elevation axes. Signature of Histogram of Orientations for Color (SHOT COLOR) extends the SHOT approach to work with texture (Salti et al., 2014). Xu et al. (2021) and Zhan et al. (2020) built a local feature descriptor based on the 3D surface normal vector and the distance between the key point and the center of gravity of its eight neighboring clusters. After creating the local feature descriptors, matching the corresponding feature descriptors is the final step for calculating the transformation matrix between two point clouds. The most common and widely used correspondence matching approach is Random Sample Consensus (RANSAC) (Fischler and Bolles, 1981). Within a limited number of iterations, it randomly selects a portion of the dataset, fits these data to a designed model, and finds the best fit based on the amount of outlying data. Rusu et al. (2009) introduced the Sample Consensus Initial Alignment (SAC-IA) as the improvement of the greedy correspondence matching algorithm. For each set of sample points in one point cloud frame, it finds a list of candidate points that have similar histograms in the other frame and finds the best match by calculating the error metric of the rigid transformation this match created. Another much faster correspondence matching approach is Fast Approximate Nearest Neighbors (FLANN) (Muja and Lowe, 2009). This approach automatically selects the nearest neighbors with a search algorithm, optimizes parameters based on search tree build-time and memory cost functions, and quickly searches the most similar correspondence candidates with the nearest neighbor search tree. More recently, end-to-end local feature-based registration processes have been constructed using neural networks (Lu et al., 2019; Li et al., 2020; Ao et al., 2021), showing significant performance improvements compared to traditional approaches (Li et al., 2020; Ao et al., 2021). While Ao et al. (2021) fit the input point cloud image to a designed cylindrical space to create point-based local key points, Lu et al. (2019) directly applied a multi-layer perceptron (MLP) model to raw point cloud images to create semantic single-point-based local feature points. Li et al. (2020) used multi-view rendering to estimate different multi-view patches from different viewpoints to ensure the point cloud registration is rotation invariant. However, these approaches suffer from common issues of noise, distortion, and errors in the point cloud data associated with local feature descriptors, which significantly affect the accuracy and performance of the algorithms, causing poor performance in dynamic, cluttered, and complex environments (Li et al., 2020; Ao et al., 2021).

### 2.2 Global 3D features

Similar to the local feature descriptor, the global feature descriptor is another traditional approach to point cloud registration with multiple variations. Madry et al. (2012) proposed encoding point clouds with Global Structure Histograms (GSH), which are formed from the distribution of 3D surface-shape characteristics found with local descriptors. More recently, the Scale Invariant Point Feature (SIPF) was proposed as a new global feature descriptor (Lin et al., 2018). First, an object or the scene is represented by encoding the border with the combination of LRF and the covariance matrix defined in the SHOT feature (Tombari et al., 2010). The SIPF value is computed as q* = arg min_*q*_‖*p* − *q*‖ between feature point *p* and the edge point *q* as the reference direction. After dividing the angle q* of local cylindrical coordinates into *N* regions, whose angle is within 2*πiN* and 2*π*(*i* + 1)*N* for *i* = 0, 1, *…*, *N* − 1, the SIPF descriptor is constructed by concatenating all the normalized cell features $D_{i}=\exp\left(\frac{d_{i}}{1-d}\right)$ , where *d*_*i*_ is the minimum distance between a point *p* and the *ith* region. Another recent global feature descriptor with promising computational time and robustness to Gaussian noise is the Global Orthographic Object Descriptor (GOOD) (Kasaei et al., 2016), which is also built based on LRF. GOOD first orthographically projects (i.e., all projection lines are orthogonal to the projection plane) onto three planes constructed as the *X*-*Z*, *X*-*Y*, and *Y*-*Z* axes, respectively, and then each plane is divided into multiple bins so that the feature is built from concatenating the entropy and variance vectors from each distribution matrix by counting the number of points for each bin. In addition, Gojcic et al. (2020) and Choy et al. (2020) used the output of the sparse tensor as the fully convolutional global geometric feature, which is efficient and discriminative. Although global features are more robust to noise and distortion, having a sufficient number of descriptors and accurate correspondence is a common challenge that hand-crafted and machine-learning global feature descriptors experience, particularly in challenging environments or with data captured from distinct perspectives. As the global feature descriptors illustrate the entire point cloud frame, the most common approach is defining an optimization function based on their feature to find the minimum error between the two global features (Lei et al., 2017; Gojcic et al., 2020).

### 2.3 No-correspondence approaches

No-correspondence approaches do not rely on calculating the transformation matrix using correspondences between described key points. Instead, the optimal transformation matrix that minimizes the projection error between the feature sets of two point cloud images is found. Huang et al. (2020a) first extracted the point-wide rotation-attentive feature using an MLP model with a max-pool layer and then calculated the transformation matrix that minimizes the feature-metric project error across all points within the images. Similarly, Le et al. (2019) constructed the transformation matrix from a list of optimization equations between two randomly selected subsets from the source and target point cloud frame and refined the transformation matrix using the iterative closest point (ICP) (Lu and Milios, 1997) algorithm. However, both approaches require a large common area between two point cloud images so that the minimum project error can be found within the overlapping point cloud, rendering them ineffective in challenging environments that can appear significantly different from different perspectives or in situations where sensors have a minimal overlapping field of view.

### 2.4 Estimation approaches

Point cloud estimation algorithms are commonly used in object modeling fields. Schaer et al. (2007) relied on the feature of laser and terrain, estimating the missing laser scanning geometric terrain data by first calculating the local normal vector and curvature of the point cloud so that all points can be pre-classified; then computing the special 3D footprint from the normal vector, laser direction, and laser beam divergence of the laser points; and finally combining all the previous information as the accumulation of random error and estimating a point based on the error and the normal of its neighborhoods. Tagliasacchi et al. (2009) proposed another point cloud estimation algorithm that inspires our approach using the symmetry of the human body. It uses the rotational symmetry axis (ROSA) algorithm, which we will further discuss in the method section, to find the center of mass (CoM) point of a layer of points. Then, based on the human symmetric feature, the missing points are estimated from the existing correspondent points with the CoM point as the reference. More recently, Huang et al. (2020b) and Wang et al. (2020) used machine learning approaches to estimate the missing points in the point cloud frame. Huang et al. (2020b) first built the skeleton-based feature point using the iterative farthest point sampling (IFPS) strategy, then trained the combined multi-layer perceptions (CMLP) with three different scales of IFPS feature points, and finally used a pyramid-like point decoder to estimate all points in the point cloud frame in different resolutions from the CMLP output feature vectors. Similarly, Wang et al. (2020) first extracted global point features using two PointNet feature extraction networks, then mapped the global point features to the categorized coarse representation of the point cloud, and finally refined the point cloud frame by generating higher resolution points based on the coarse point cloud. As all the aforementioned estimation approaches rely on some features or knowledge of the object, the accuracy of the result cannot be guaranteed, especially with errors and distortion (Schaer et al., 2007; Wang et al., 2020). Therefore, in challenging indoor environments, which are environments that our algorithm is targeting, existing completion approaches, whether registration or estimation, are insufficient. Inspired by the previous work of Czarnuch and Ploughman (2014), our objective is to develop an approach that can create a more comprehensive and complete point cloud in these challenging environments using data provided by multiple perspective-independent sensors. Specifically, we propose a novel approach using a unique spatiotemporal feature based on human motion trajectories referenced to the known ground plane, leveraging the strengths of both registration and estimation approaches for indoor point cloud completion.

## 3 Methodology

**FIGURE 1:**
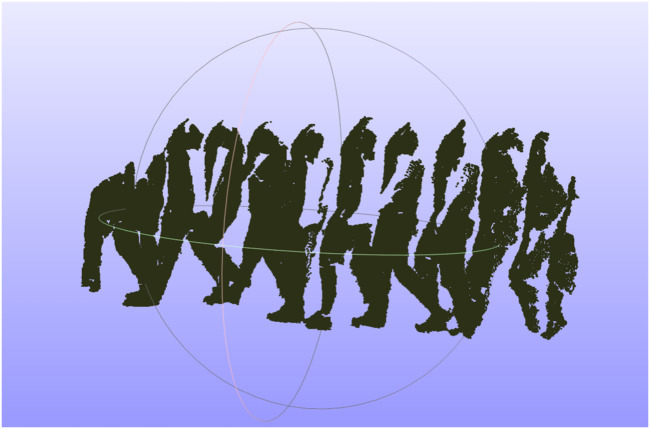
Human walking path created from a series of human point clouds with intervals of 10 frames.

Our novel point cloud completion approach first utilizes the work of Zhang and Czarnuch (2020) to reduce the complexity of the 3D point cloud problem. Two arbitrary point clouds must be aligned in six degrees of freedom: three rotations and three translations. By detecting and aligning to the ground plane, the problem space is reduced to three degrees of freedom: one rotation around the ground plane surface normal and two translations parallel to the ground plane. In Zhang and Czarnuch’s (2020) algorithm, the ground plane is estimated from a series of 3D point cloud images captured from an unknown arbitrary RGB-D camera perspective with the assumption that at least one person is visible and is walking in the cameras’ FOV. The algorithm first extracts and stores all the largest planes *P*_*i*_ from the 3D point cloud image as potential ground plane candidates. After removing all the largest planes in the point cloud, all isolated clusters of points are labeled as potential moving human candidates *O*_*i*_. Then, the corresponding 2D RGB image is described with Scale-Invariant Feature Transform (SIFT) features, constructing moving trajectory vectors $\vec{t}$ for each moving cluster within a block of *n* = 5 frames *B*_*i*_, and identifying moving human(s) *H*_*i*_ among the potential cluster candidates *O*_*i*_ using Motion-Split-And-Merge (MSAM) (Dragon et al., 2012) within each block. Finally, the ground plane is estimated after applying a cascaded filter to the ground plane candidates based on the geometric relationship between the ground plane and all the objects *O*_*i*_ and the trajectories of identified humans *H*_*i*_ in the point cloud. The output of the previous ground plane estimation algorithm includes a parametric model of the ground plane *GP*, the point cloud(s) of the moving human(s) *H*_*i*_ and associated trajectory vectors $\vec{t_{i}^{k}}$ , the isolated point clusters *O*_*i*_ that represent static objects in the room, and the roll *R*_*r*_ and pitch *R*_*p*_ angles of the camera. By applying the ground plane estimation algorithm to the outputs of pairs of perspective-independent 3D sensors, we can obtain information regarding the ground plane position, the position(s) and direction(s) of moving human(s), the roll and pitch orientation angle of both cameras relative to the ground plane, and the height (*y*-axis) of the camera from the ground plane. With the assumption that at least one moving human walks through a common area between the two sensors’ FOVs, we can align the 3D outputs of these two sensors based on the ground plane that determines the rotation around the *x*-axis and *z*-axis and the translation information between the two sensors along the *y*-axis in the 3D registration transformation matrix. Hence, by first aligning two point clouds to their common ground planes, the 3D registration problem between two arbitrarily located sensors is simplified to a 2D problem with three unknowns remaining: the translation along the *x*-axis and *z*-axis and the rotation angle around the *y*-axis. In addition, by converting this 3D registration to a 2D problem, all the points and vectors in 3D space are reduced to 2D points and vectors by removing the *y* value from the original 3D space. For calculating the remaining three unknowns (*x*-axis and *z*-axis translations and *y*-axis rotation) in the transformation matrix, our approach is inspired by Czarnuch and Ploughman (2014), which targeted point cloud registration specifically in indoor environments that are highly cluttered, dynamic, and complex and contained a moving human. Czarnuch and Ploughman (2014) first segmented the human using depth-based background subtraction and then calculated the CoM of the walking human for each frame in a sequence of frames captured by two sensors. Then, they registered the point clouds of the two 3D sensors by spatially aligning the walking paths calculated from the two point clouds. This new type of feature preserved the spatial trajectory in both point clouds and supported registration in conditions where a large rotation and translation angle existed between two sensors. However, the approach required an existing background model, did not utilize the temporal information provided by identifying the CoM in sequential frames from synchronized sensor pairs, and required a nonlinear walking path for successful registration. We aimed to perform a similar final registration using the 3D trajectories of all moving humans. Improving on Czarnuch and Ploughman (2014)’s process, we used the extracted clusters that represented humans from our ground plane detection (Zhang and Czarnuch, 2020) and found the center of mass of each cluster to construct the walking paths of any number of humans, overcoming some of the limitations of Czarnuch and Ploughman (2014). Notably, the ground plane estimation algorithm does not guarantee that the moving human(s) and the ground plane are found in every block of frames, suggesting that our temporal trajectory vector may be missing some CoM data points. Figure 1 shows a sample point cloud representation of a human walking path created from 100 frames with an interval of 10 frames.

**FIGURE 2:**
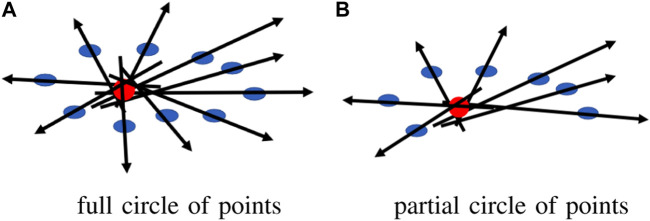
ROSA point for a circle of points **(A)** full circle of points, **(B)** partial circle of points.

**FIGURE 3:**
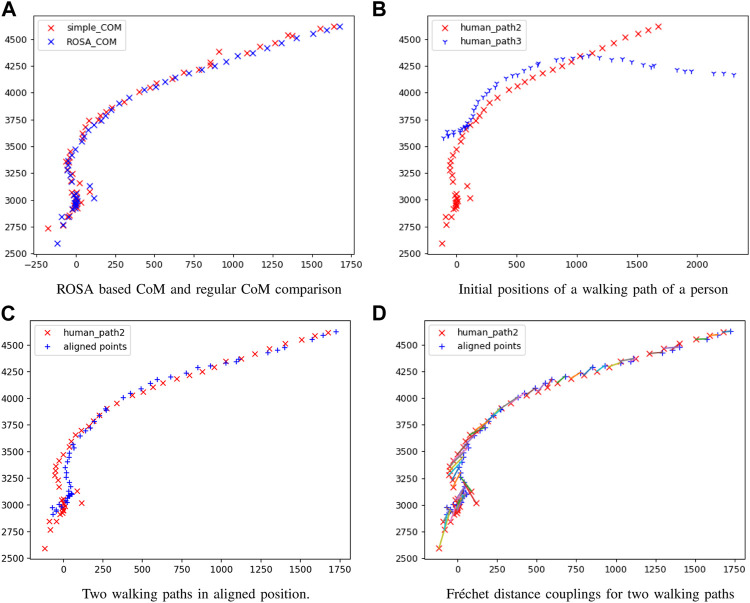
Registration results comparison **(A)** ROSA based CoM and regular CoM comparison, **(B)** initial positions of a walking path of a person, **(C)** two walking paths in aligned position, **(D)** Fréchet distance couplings for two walking paths.

The accuracy of each CoM is significantly affected by the viewing angle of each sensor. Each sensor creates a different, incomplete human point cloud based on its viewing perspective, and the CoM is calculated based on the partial point cloud that may generate considerable shifts in the estimated CoM, causing errors in the transformation matrix for point cloud registration. In addition, human clusters may contain points from objects that were incorrectly segmented along with the human, potentially introducing error in the CoM if it is directly calculated from the clusters. To improve the accuracy of our CoM calculation, we first estimated a complete human point cloud using ROSA (Tagliasacchi et al., 2009). This estimation approach utilizes the fact that the tracked object is a human, and we specifically exploit the symmetric feature of the cluster from an overhead perspective because we know the human is walking along the ground plane. The ROSA point is calculated by minimizing the sum of the squared distance to the normal vector extensions for a circle of the points, as shown in Figure 2A. By using this approach, the center of a symmetric object is accurately estimated even in conditions where a substantial portion of an object’s points are missing, similar to our condition with a partial human, as shown in Figure 2B. Figure 3 shows the differences between the ROSA-based COM points and the regular COM points, which are calculated by averaging the *x* and *z* values of all points. It is easy to see that the results of ROSA CoM points represent a smoother walking path that is closer to the real path. To implement ROSA, we first use *k*-d tree-based normal estimation (Pauly, 2003) to create a 3D normal vector for each point in the partial human point cloud. Then, for every 50 mm, we slice the human point cloud and corresponding normal vectors into multiple layers parallel to the ground plane based on the height of the human point cloud relative to the ground plane. Next, we apply ROSA to each slice to find the CoM of each slice. Finally, we generate the CoM of the human based on the average CoM of all slices rather than directly from the point cloud because we are only concerned with translations along the *x*-axis and *z*-axis and a rotation around the *y*-axis (i.e., from an overhead perspective). With this estimation approach, our CoM algorithm substantially reduces the error introduced by missing data associated with partial point clouds due to camera perspective. Figure 3 shows the estimated 3D CoM points of a single person projected onto the *x* − *z* plane from data captured from two sensors in different locations. As the walking paths for each person in both cameras and the approximate trajectory vector for each human are now known, we determine the human correspondences in both cameras by calculating the Fréchet distance (Har-Peled et al., 2002), which essentially determines the shape similarity between two point sets by calculating the minimum length of coupling from an ordered sequence of distinct pairs of vertices. The Fréchet distance is calculated according to the following equation:

$$F\left(A,B\right)=\underset{\alpha ,\beta }{\inf}\underset{t\in \left[0,1\right]}{\max}\left\{d\left(A\left(\alpha \left(t\right)\right),B\left(\beta \left(t\right)\right)\right)\right\},$$ (1)

where *d* is the Euclidean distance, *A* and *B* represent the two walking paths, and *α* and *β* indicate the parameterized function of the two paths. We first take the minimum length of the walking paths within the same time frame required by the parameterized functions, and then we find the walking path pairs (between cameras) that have the shortest Fréchet distance (Figure 3) to identify all moving object paths that possibly represent the same person. Finally, we apply the 2D ICP (Lu and Milios, 1997) algorithm to find the 2D rotation angle, *x*-axis and *y*-axis translations that correspond to the yaw angle rotation, the translations along the *x*-axis and *z*-axis in the 3D transformation, respectively, for each pair of walking paths, and aligning two paths based on the 2D transformation matrix, exemplified in Figure 3. The final transformation matrix from registering the walking paths is generated by calculating the 2D ICP result for all pairs of walking paths within a period of time (e.g., if more than one person is visible) and then combining them with the existing roll angle, pitch angle, and *y* translation, which is generated from the ground plane estimation, to formalize the six unknown parameters of the transformation matrix (e.g., *x*, *y*, and *z* translations and roll, pitch, and yaw).

As the walking paths are constructed from the estimated CoM and given that individual CoM points may be missing, the paths will always contain some noise and error. Accordingly, the transformation matrix that our walking path registration generates will also contain some errors. Therefore, the accuracy of our final registration is highly dependent on the accuracy of the walking path CoM estimation.

## 4 Experiments

We evaluated our algorithm based on the only suitable public dataset available (Vaquette et al., 2017), which included data from three statically mounted sensors with known locations and relative positions and one person moving within the scene. Our criteria were that a human was visible walking within the FoV of each pair of sensors that provided combined depth and RGB data at some point in time. We augmented these public data with our own captured dataset, with the intention of producing scenarios that were more complicated and more diverse, including more varied sensor locations, more persons visible in the scene at the same time, and more challenging environments. We first used a public dataset to verify the basic performance of our algorithm, and then we used our more challenging dataset to evaluate the reliability and accuracy of the algorithm more generally. In this way, we ensured our algorithm was generalizable and robust and did not rely on any artificial factors that could be introduced in our own captured dataset. Furthermore, as other datasets meeting our criteria do not publicly exist, we will make our datasets available publicly for independent verification of our results and promoting future research in related areas.

### 4.1 Public dataset

**FIGURE 4:**
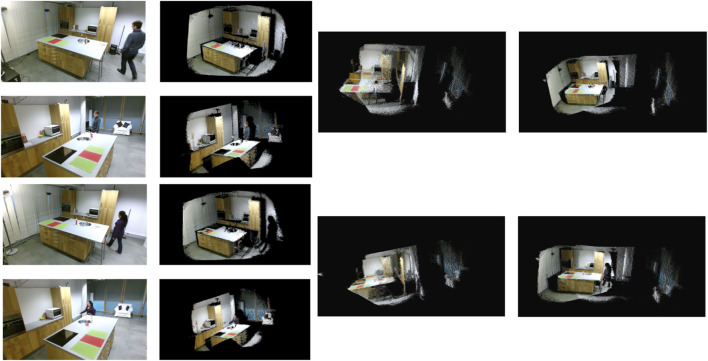
Examples of point cloud completion using the DAHLIA public dataset. The first column shows the raw RGB images, the second column is the corresponding colored point cloud for the frame, the third column shows the initial position of two point cloud frames when aligned to the ground planes and centered over each other, and the final column demonstrates the completed point cloud.

The novelty of the challenge we are trying to resolve, point cloud completion in challenging environments, and specifically our requirement for human motion, makes it difficult to use the most popular public datasets to verify the performance of our algorithm. Notably, this is because sensors are almost always ideally placed in known locations, and scene changes are incremental, allowing traditional point cloud completion approaches to be used. Therefore, the only public dataset that meets all of our requirements (e.g., the data are captured from multiple RGB-D cameras and at least one human can be seen moving in the common field of view of all pairs of sensors at some point) is the DAily Human Life Activity (DAHLIA) public dataset (Vaquette et al., 2017). This public dataset is captured from three static Microsoft Kinect 2 sensors, which are placed at 1.85 m above the ground and 18-degree pitch angle to form a right triangle. It includes segments where, at most, a single person can be seen walking around in a room while performing different daily activities. A total of 51 data trials are available, and each trial is approximately 50 s long, stored as sequential RGB and depth image pairs. In addition, the ground truth data for each camera’s roll, pitch, and yaw angles and relative positions are fully documented, allowing for a simple calculation of the true transformation required to align the data between the three pairs of sensors. The only variability in this public dataset is that different persons walk into the scene and perform varied daily activities, so we only selected one data trial for each unique person. In other words, nothing else in the environment (e.g., objects and sensor locations) changes over the entire dataset. Since the trials do not vary significantly outside person and activity, we selected the first twenty trials with different humans for evaluation and randomly selected two out of three sensors for each trial. Notably, all data share a similar human walking path, and data were missing for trial 09, which resulted in 19 registration data trials for our evaluation. Figure 4 shows an example of the original data and the registration result of one trial. However, as only one single person walks in the scene at any time and camera perspectives are never varied in different data trials, this public dataset is only used to verify the basic performance and functionality of our algorithm. The parameters of the 2D ICP we used are a maximum of 100 iterations, a 15-pixel searching distance threshold, one-pixel translation coverage, one-degree rotation coverage, and at least half of the total points as inliers. Out of 19 different trials, each with a different human participant, we successfully registered the walking paths in all 19 trials and completed the point clouds, with the registration errors shown in Table 1 relative to the documented ground truth. The rotation errors are calculated using the angular difference between the registered rotation angle and the ground truth angle divided by 180 degrees along the roll, pitch, and yaw rotations. The translation errors are calculated as the difference in distance between the registered translation and the ground truth translation divided by the smaller point cloud bounding box length along each *x*-, *y*-, and *z*-axis to provide a normalized measure. Overall, the largest rotation error was 10.75% along the yaw axis. Notably, the average yaw rotation error was 5.62%, over double the average rotation errors around the roll (1.91%) and pitch (2.28%) axes. This is mainly because the roll and pitch axes are calculated based on aligning the ground plane, whereas the yaw rotation is created based on aligning the walking path, which contains more noise when the human is close to other objects in the room and the number of frames (i.e., time) that the pairs of 3D cameras have a clear view of the isolated human, which is very short in the public dataset. The translation errors were more consistent than the rotation errors across the *x*-, *y*-, and *z*-axis, averaging 2.96%, 2.92%, and 2.44%, respectively. Notably, the minimum errors approached zero (i.e., perfect registration), and the largest errors were spread across four different trials: S08_A3, S11_A2, S15_A1, and S16_A1.

**TABLE 1:** DAHLIA public dataset detailed results.

	Rotation error in %			Translation error in %		
Trial name	Roll	Pitch	Yaw	*x*	*Y*	*z*
S01_A1	**0.001**	1.689	**0.002**	1.856	0.340	0.208
S02_A2	0.865	2.845	7.573	1.798	3.201	1.790
S03_A2	1.138	1.006	4.435	3.212	4.854	0.469
S04_A2	1.417	4.410	5.153	1.232	4.394	4.951
S05_A1	2.611	**0.192**	4.757	3.776	1.255	0.911
S06_A2	0.761	2.537	5.910	**0.617**	4.855	4.338
S07_A2	3.286	1.217	4.110	3.209	2.729	1.177
S08_A3	**4.276**	3.982	7.608	**8.847**	**0.204**	3.403
S10_A1	1.291	2.327	3.043	1.410	0.605	3.192
S11_A2	2.281	4.986	**10.754**	6.073	**8.440**	2.861
S12_A1	2.522	0.440	2.044	3.588	1.757	0.267
S13_A1	2.407	0.795	8.085	1.623	1.897	0.530
S14_A1	0.577	4.164	8.124	3.506	4.992	3.203
S15_A1	0.852	**6.689**	9.847	2.284	6.927	**0.144**
S16_A1	0.415	4.287	9.225	1.396	1.460	**5.424**
S17_A1	2.801	0.970	2.507	0.944	1.220	4.560
S18_A1	1.744	0.663	4.144	4.935	1.960	1.226
S19_A1	4.062	0.522	4.661	1.981	2.666	4.203
S20_A1	3.044	1.219	3.103	3.825	1.697	3.412
Average	1.913	2.276	5.619	2.958	2.919	2.435
Maximum	4.276	6.6893	10.754	8.847	8.440	5.424
Minimum	0.001	0.192	0.002	0.617	0.204	0.144

The bold values in each column indicate the highest and the lowest error among the different datasets.

### 4.2 Private dataset

The public dataset scenarios did not represent varied and diverse camera positions and perspectives, unique walking paths, or dynamic and varied environments. To complement the public dataset, we captured data that were more representative of challenging real-world scenarios, including a large variety of camera orientations, significantly unique camera locations, small overlapping FOV between sensor pairs, complex occupant paths, and varied human activities. To test the performance of our algorithm under these more challenging environments, we captured seven data trials using two Azure Kinect sensors and the registration steps with the same parameters as the public dataset. Within the data trails, our two sensors had a 90-degree roll angle with different yaw angles in D01 and D02. Our two sensors had a 180-degree relative roll angle with different yaw and pitch angles in D03, D04, and D05. Our two sensors had 180-degree yaw relative angles (facing each other) in D06 and 90-degree pitch angles relative to each other in D07. Each data trial was captured using the Azure Kinect MKV recorder (Azure kinect dk recorder, 2020) and stored as a *mkv* video file, which contained the synchronized color and depth frames, as well as the sensor metadata (intrinsic and extrinsic parameters).

**FIGURE 5:**
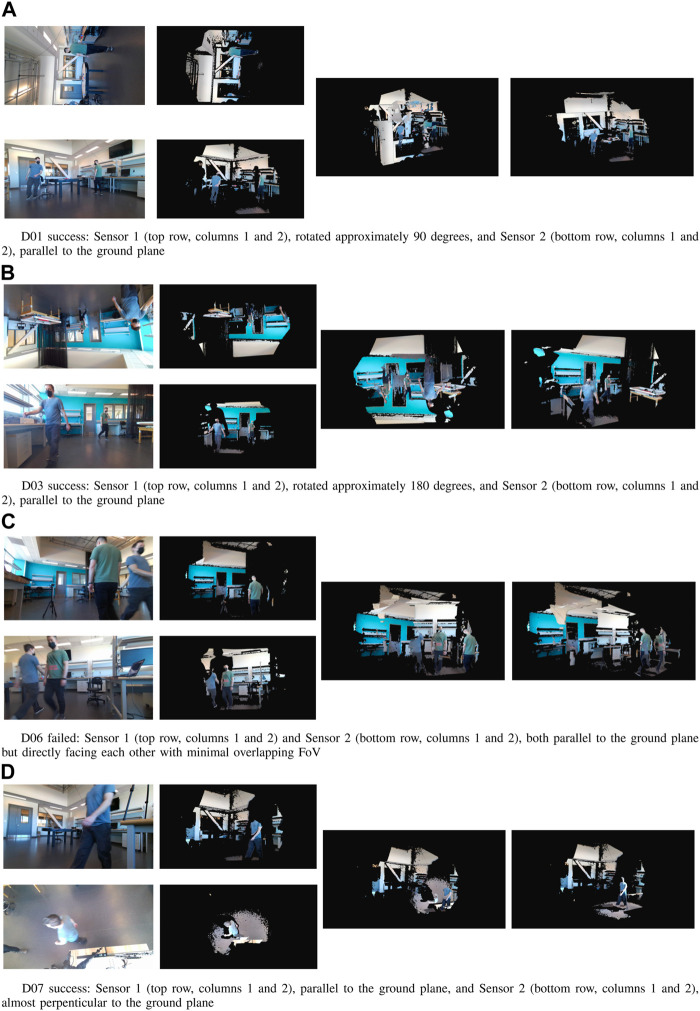
Examples of point cloud completion using our collected dataset. **(A)** D01 success: Sensor 1 (top row, columns 1 and 2), rotated approximately 90 degrees, and Sensor 2 (bottom row, columns 1 and 2), parallel to the ground plane, **(B)** D03 success: Sensor 1 (top row, col-umns 1 and 2), rotated approximately 180 degrees, and Sensor 2 (bottom row, columns 1 and 2), paral-lel to the ground plane, **(C)**, D06 failed: Sensor 1 (top row, columns 1 and 2) and Sensor 2 (bottom row, columns 1 and 2), both parallel to the ground plane but directly facing each other with minimal overlap-ping FoV, **(D)**, D07 success: Sensor 1 (top row, columns 1 and 2), parallel to the ground plane, and Sensor 2 (bottom row, columns 1 and 2), almost perpenticular to the ground plane.

Figure 5 exemplifies some of the unique conditions we created to evaluate our approach. Notably, the public dataset was captured using the Microsoft Kinect v2 sensor, which does not provide any synchronization between sensors, and the sensors themselves do not guarantee that the data will be available for each frame. The Azure Kinect sensor, in comparison, has built-in synchronization, which is configurable and results in much more reliable synchronization (on the order of milliseconds). In addition, the highest RGB image resolution of the Azure Kinect sensor is 4,096 × 3,072 ( ∼12 million points after mapping each RGB pixel to point cloud point), whereas the Kinect v2 sensor only provides an RGB resolution of 1,920 × 1,080 ( ∼2 million points) (Azure kinect and kinect windows v2 comparison, 2022). Hence, our approach can be expected to have higher accuracy with our private dataset than with the public dataset, as shown in Table 2, when excluding trial D06. In trial D06 [shown in 6(b)], the two sensors were facing each other with a very small overlapping field of view, and two participants were walking in straight lines close to each other, resulting in very short common overlapping walking paths. The original alignment to the ground plane in this trial was notably very good (i.e., roll, pitch, and translation along the *y*-axis). However, the final completion was poor, with a 93.39% error in the yaw angle and a translation error of 87.33% and 2.42% along the *x*- and *z*-axis. Without this very challenging trial, the average error of our own data on *yaw* and *x*- and *y*-axis is excellent, even slightly lower than the less challenging public dataset. Notably, compared to the public dataset, our trials had significantly smaller ground planes, more complex occupant configurations, and walking paths, and the actual values of the three unknowns (i.e., roll, pitch, and translation along the *y*-axis) in our own datasets are substantially larger. Despite these challenging conditions, all pairs of walking paths in our own data trials converged using ICP within 100 iterations with the same parameters, which means that the 2D ICP could not distinguish the difference between the proper registration and error.

**TABLE 2:** Private dataset detailed results.

	Rotation error in %			Translation error in %		
Trial name	Roll	Pitch	Yaw	*x*	*y*	*z*
D01	0.9359	4.6464	2.3321	3.8726	0.0457	2.5682
D02	3.2414	2.5388	0.6644	3.1797	2.2609	2.0714
D03	4.8244	1.5742	0.6865	2.3831	7.7013	3.4017
D04	4.8333	0.5910	1.9453	0.6631	2.2753	3.2261
D05	0.871	2.8698	1.0831	2.8161	5.0866	2.4757
D06	0.1977	3.0858	93.3972	87.3272	4.4736	2.4185
D07	1.5906	2.7279	0.2058	2.3706	1.8662	0.8621
Average	2.3403	2.5762	14.3306	15.2303	3.3871	2.4319
No D06 ave	2.7161	2.49135	1.1528	2.5475	3.206	2.4342

### 4.3 Registration algorithm comparison

**FIGURE 6:**
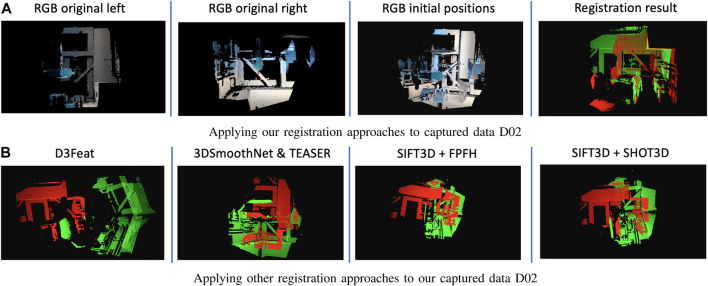
Registration results comparison. **(A)** Applying our registration approaches to captured data D02, **(B)** Applying other registration approaches to our captured data D02.

We selected three popular machine learning-based registration approaches that provided pre-trained models and registration scripts to evaluate the objective performance of our approach. We further evaluated our approach against two feature-based approaches. We used all three machine learning models as defined with our private dataset. The D3Feat (Bai et al., 2020) approach resulted in incorrect transformation matrices, whereas both 3DRegNet (Pais et al., 2019) and 3DSmoothNet with TEASER (Yang et al., 2020) could not find sufficient correspondences between the point cloud pairs. Similarly, the feature-based approaches based on SIFT3D with FPFH (Rusu et al., 2009) and SIFT3D with SHOT3D (Salti et al., 2014) also could not find acceptable transformation matrices due to an insufficient number of correspondences. Results that use these approaches are shown in Figure 6.

## 5 Discussion and conclusion

In this study, we proposed a novel point cloud completion approach that can combine the 3D point clouds captured from multiple RGB-D sensors placed in unknown positions and perspectives in challenging environments into a complete point cloud, with the minimal assumption of at least one person visibly moving within the common FoV of sensor pairs. Our algorithm eliminates the instability and noise introduced by processing the point clouds captured in challenging indoor environments with large 3D sensor perspective differences, where existing registration algorithms encounter limitations and issues (e.g., poor feature description or failed correspondence estimation) or require overly restrictive preconditions or assumptions (e.g., the ground plane at the bottom of the scene or small perspective difference between sensor pairs). We successfully completed the point clouds for 25 of 26 trials (96.2%), including data from both a public dataset and our own more challenging data. The only scenario that caused our algorithm to fail was a scene where two sensors faced each other, and two humans walked along two parallel, straight lines in opposite directions. In this challenging scenario, for each sensor, the person on the left was correctly identified as walking toward the sensor, and the person on the right was identified as walking away from the sensor. However, the resulting Fréchet distance calculations could not differentiate between the two walking paths, resulting in incorrect matching. However, this scenario is arguably challenging enough that even a human may struggle to accurately align the point clouds based on spatial normalization and activation labeling (Brett et al., 2002), suggesting that some challenging conditions are potentially impossible to automatically align.

We first located the ground planes in each set of sensors by finding all moving objects, aligned pairs of point clouds to the common ground plane, and identified all moving humans in the scene. This reduced the complexity of the 3D point cloud completion challenge from six unknowns (translation along the *x*-, *y*-, and *z*-axis and roll, pitch, and yaw rotations) to three unknowns (translations along the *x*- and *z*-axis and yaw rotation). For each human reliably tracked in each sensor, we built a walking path using the ROSA estimation technique, which generated a reliable CoM vector array independent of the perspective of the capturing sensor. We achieved this perspective independence by utilizing the ground plane information to convert the 3D CoM walking path to a 2D path from a sensor perspective parallel to the surface normal of the ground plane (i.e., overhead perspective). We used this 2D walking trajectory as a time-series feature descriptor, with a discriminative and descriptive power proportional to the complexity of the path. In other words, the more complex a walking path was, the more uniqueness this feature had, which led to a more accurate transformation matrix calculation. Finally, we used the Fréchet distance to find corresponding walking paths and calculated the registration transformation matrix between the corresponding paths using 2D ICP. Since we targeted challenging environments where pairs of sensors could have significant perspective differences, our algorithm did not include a final alignment after 2D ICP registration. We evaluated our approach using one public dataset, which contained 19 trials with different persons in each data trial, and our own purposefully captured data, which included seven unique and challenging scenarios (e.g., large perspective differences, small common FOV, more than one walking person, and unique walking patterns). Figure 6 shows that our algorithm is robust against perspective variation and is reliable in challenging indoor environments, where other approaches could not calculate a low-error transformation matrix with state-of-the-art features or pre-trained models. Our approach notably has three major limitations. First, the performance of the registration result only depends on the accuracy of 2D ICP between two walking paths because no final refinement is applied after the initial alignment. Therefore, this approach is mainly targeting point cloud registration under common yet challenging scenarios that may be difficult for even a human to complete, but it may not be suitable for applications that require highly accurate point cloud completion. Our algorithm also only considers the walking path as a feature; therefore, it requires sufficiently unique paths to succeed and can fail in scenarios such as face-to-face cameras where two people walk toward each other in parallel paths perpendicular to the camera lens surfaces. Our approach also relies on the Fréchet distance to differentiate between 2D walking paths, so people that have similar body shapes and walk in similar walking paths at the same time will result in identical features in our algorithm. In the future, we will focus on addressing the limitations we have identified in our current algorithm, including testing our algorithm on a larger number of more complex and custom scenarios.
